# The Social Maelstrom—New York

**Published:** 1910-05

**Authors:** 


### The Social Maelstrom—New York.

After the first Thaw trial, when Delmas, of California, was Thaw's lawyer, the Lamb Club gave him a dinner on the eve of his departure for San Francisco. A toast to New York was proposed, and Delmas was called on for a speech. He said:

"Vulgar of manner, over-fed, over-dressed and underbred,
Heartless, Godless, hell's delight; rude by day and lewd by night;
Ruled by man and large the brute, ruled by Jew and prostitute;
Purple-robed and pauper-clad, rotten, raving, money-mad;
A squirming herd in money's mesh, a wilderness of human flesh,
Crazed by avarice, lust and rum—New York, thy name is
Delirium."

